# The relationship between structural analysis of the hand and clinical characteristics in psoriatic arthritis

**DOI:** 10.1038/s41598-022-23555-5

**Published:** 2022-11-07

**Authors:** Alexander Pfeil, Marcus Heinz, Tobias Hoffmann, Tobias Weise, Diane M. Renz, Marcus Franz, Ansgar Malich, Dominik Driesch, Peter Oelzner, Gunter Wolf, Joachim Böttcher

**Affiliations:** 1 Department of Internal Medicine III, Jena University Hospital, Friedrich Schiller University Jena, Am Klinikum 1, 07747 Jena, Germany; 2 BioControl Jena, Hans-Knöll-Straße 6, 07745 Jena, Germany; 3 Department of Pediatric Radiology, Hannover Medical School, Institute of Diagnostic and Interventional Radiology, Carl-Neuberg-Str. 1, 30625 Hannover, Germany; 4 Department of Internal Medicine I, Jena University Hospital, Friedrich Schiller University Jena, Am Klinikum 1, 07747 Jena, Germany; 5 Institute of Diagnostic Radiology, Suedharz-Hospital Nordhausen, Dr.-Robert-Koch-Straße 38, 99734 Nordhausen, Germany

**Keywords:** Musculoskeletal system, Osteoimmunology, Rheumatic diseases

## Abstract

Up to now, there is only limited information available on a possible relationship between clinical characteristics and the mineralization of metacarpal bones and finger joint space distance (JSD) in patients with psoriatic arthritis (PsA). Computerized digital imaging techniques like digital X-ray radiogrammetry (DXR) and computer-aided joint space analysis (CAJSA) have significantly improved the structural analysis of hand radiographs and facilitate the recognition of radiographic damage. The objective of this study was to evaluate clinical features which potentially influence periarticular mineralization of the metacarpal bones and finger JSD in PsA-patients. 201 patients with PsA underwent computerized measurements of the metacarpal bone mineral density (BMD) with DXR and JSD of all finger joints by CAJSA. DXR-BMD and JSD were compared with clinical features such as age and sex, disease duration, C-reactive protein (CRP) as well as treatment with prednisone and disease-modifying antirheumatic drugs (DMARDs). A longer disease duration and an elevated CRP value were associated with a significant reduction of DXR-BMD, whereas JSD-parameters were not affected by both parameters. DXR-BMD was significantly reduced in the prednisone group (–0.0383 g/cm²), but prednisone showed no impact on finger JSD. Patients under the treatment with bDMARDs presented significant lower DXR-BMD (–0.380 g/cm²), JSD_MCP_ (–0.0179 cm), and JSD_PIP_ (–0.0121 cm) values. Metacarpal BMD was influenced by inflammatory activity, prednisone use, and DMARDs. In contrast, finger JSD showed only a change compared to baseline therapy. Therefore, metacarpal BMD as well as finger JSD represent radiographic destruction under different aspects.

## Introduction

Psoriatic arthritis (PsA) is a multisystemic and chronic inflammatory musculoskeletal disease associated with cutaneous psoriasis, affecting most commonly the axial skeleton as well as peripheral joints of hands and feet^1,2^.

Conventional radiology has been the gold standard assessing the damage and radiographic progression in PsA^2,3^. As shown by Siannis et al., in the majority of patients radiological damage is detected before clinical damage is observed^4^.

Computer-aided image analysis is increasingly utilized within radiology, connecting elements of artificial intelligence and computer vision with radiological image processing. Computer-based structural analysis of hand radiographs include digital X-ray radiogrammetry (DXR) and computer-aided joint space analysis (CAJSA). These diagnostic techniques allow the quantification of bone mineral density (BMD) and finger joint space distance (JSD) in rheumatoid arthritis (RA) and PsA^5–7^. Therefore, DXR- and CAJSA-parameters serve as surrogate markers for radiological and structural damage as well as for radiological progression in RA^5,8^.

Some recent published studies revealed a demineralization of the metacarpal bones in PsA and BMD as an additional hallmark of radiographic damage in PsA^6,9,10^. In addition, joint space narrowing of finger joints represents a characteristic radiologic feature of the PsA-associated joint destruction process^2^.

So far, there is little information available regarding the relationship between clinical attributes and the mineralization of the metacarpal bones and finger JSD in patients with PsA. Therefore, the aim of our retrospective study was to evaluate clinical parameters which potentially influence periarticular mineralization of metacarpal bones as well as finger JSD in these patients.

## Methods

The study enrolled 201 PsA-patients. The following parameters were captured as clinical features: Disease duration, swollen joint count (SJC), tender joint count (TJC) inflammatory activity measured by C-reactive protein (CRP) and Disease Activity Score 28 (DAS28) as well as therapy with prednisone, non-steroidal anti-inflammatory drugs (NSAIDs), coxibs and disease-modifying antirheumatic drugs (DMARDs). Data of SJC, TJC and DAS28 were available for 58 patients. All subjects underwent radiographic examinations of the hands (anteroposterior view).

### Computer-based structural analysis (Fig. 1)

**Figure 1:**
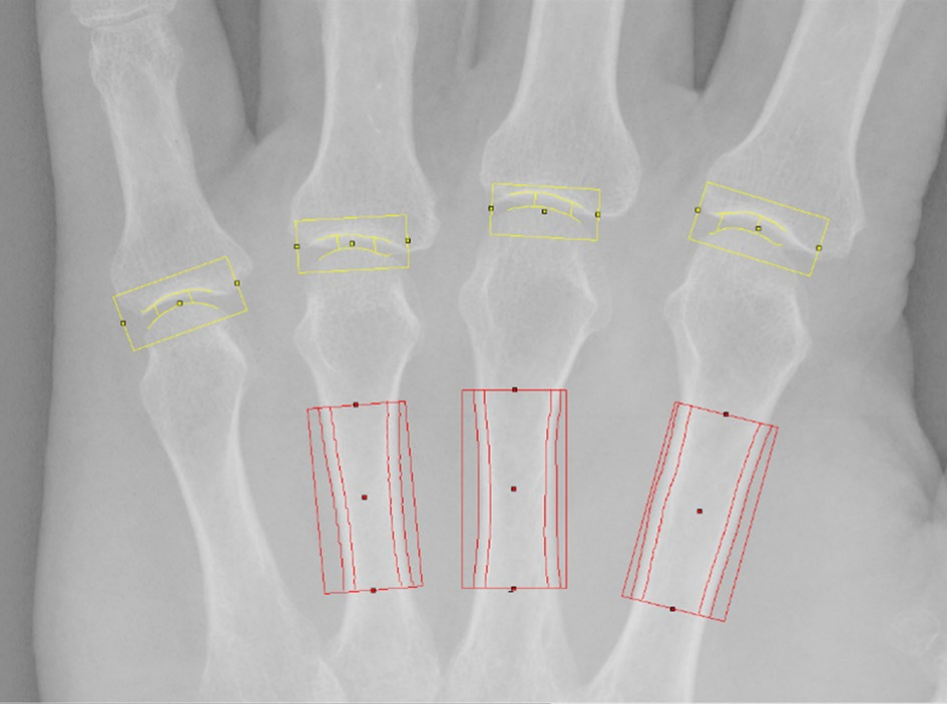
Computer-based structural analysis using digital X-ray radiogrammetry (DXR) for the quantification of metacarpal bone mineralization (region of interest: red box) and computer-aided joint space analysis (CAJSA) for the quantification of finger joint space distance (region of interest: yellow box).

Computer-based structural analysis of the hands included DXR for the quantification of metacarpal bone mineralization and CAJSA for the quantification of finger JSD.

### Digital X-ray radiogrammetry

DXR (Pronosco X-Posure System™, Version 2.0; Sectra; Sweden) was applied to determine BMD (g/cm^2^)^11^. All plain radiographs were subsequently scanned (Scanner UMAX Power Look 1100, resolution 300 dots per inch) into the DXR system, turning them into digital images.

The system performs a continuous self-testing validation to maintain the quality of digital X-ray imaging, stopping the process when imaging becomes inferior (i.e. incorrect determination of bone contours and false identification of bone structures). The computer algorithms automatically defined regions of interest around the narrowest bone parts of the metacarpals II, III and IV and subsequently determined the outer and inner cortical edges of the cortical metacarpal bone parts and bone mineral density of the metacarpal bones (DXR-BMD) was verified.

### Computer-aided joint space analysis

CAJSA (Radiogrammetry Kit, Version 1.3.6; Sectra; Sweden) measured all JSD_MCP_ (metacarpophalangeal joints, thumb to small finger), JSD_PIP_ (proximal interphalangeal joints, index finger to small finger), and JSD_DIP_ (distal interphalangeal joints, index finger to small finger). The measurement procedure was performed as follows: positioning of the region of interest to mark the particular joint to be measured. This is the only operator-dependent procedure in the entire measurement process. The CAJSA software is based on an automatic edge filtering within the region of interest identifying the specified joints. A 1.5 cm long edge across each bone was further determined and the distance between the two edges estimated as a function of the horizontal position. This was followed by calculation of the mean average and standard deviation of the distance over an extended interval of 0.8 cm by the CAJSA software. The distance between the bones was defined to be the edge interval for which the standard deviation is minimal. Additional, the Z-score as an age- and sex-independent parameter for the quantification of finger JSD was quantified^12^.

### Statistical analysis

Statistical computations were performed, using the programming language Python (version 3.6.9) and the additional packages NumPy (version 1.16.2), pandas (version 0.25.0), and Statsmodels (version 0.11.1). Data visualisation was carried out using the packages Matplotlib (version 3.3.0) and Seaborn (version 0.9.0).

The results of the DXR- and CAJSA-measurements were expressed as mean and standard deviation.

To adjust for age and sex-related changes in finger JSD that are disease-independent, Z-scores of all finger JSD were calculated according to Pfeil et al. 2009^12^ as followed:

$${\text{JSD}} - {\text{MCP}}_{{{\text{patient}}}} {-}{\text{ JSD}} - {\text{MCP}}_{{\text{age and sex matched control}}} /{\text{standard deviation }}\left( {{\text{SD}}} \right)_{{\text{age and sex matched control}}}$$

To adjust for age- and sex-related changes in BMD as well as disease-associated finger JSD, linear regressions models (statsmodels.formula.api.ols) were adjusted for DXR-BMD and all finger JSDs as well as their Z-scores as dependent variables (y_i), using age and sex as independent variables (Eq. 1).

1 $${y}_{i}= {\beta }_{0,i} + {\beta }_{1,i}*age + {\beta }_{2,i}*sex$$

The respective residuals of these models were used as dependent variables (y_resid_i) in linear regression models for SJC, TJC, CPR, DAS28, disease duration, prednisone and DMARDs treatment as independent variables (x_j). Furthermore, all Z-scores were adjusted for disease-associated age and sex-related effects accordingly (Eq. 2).

2 $${y}_{resid,i}= {\beta }_{0,i} + {\beta }_{1,i}* {x}_{j}$$

To stabilize variance and compensate skewness, the continuous variables CRP and disease duration were log10 transformed prior to linear regression. Since disease duration included zeros, an off set of one year was added to disease duration before log10 transformation.

Anova Type II (statsmodels.stats.anova.anova_lm) was used for all linear regression models. P-values were reported to describe the influence of the respective independent variable on the respective dependent variable. P-values < 0.05 were considered as significant results.

### Ethics approval

All examinations were performed in accordance with the rules and regulations of the local human research and Ethics Committee. The study protocol was approved by the Ethics Committee of the Friedrich-Schiller-University Jena, Germany (registration number 2018-1212). A consent was obtained from all subjects and/or their legal guardian(s). As a special note, the authors emphasize that all radiographs used for DXR- and CAJSA-calculations were performed as part of routine clinical care; no additional radiographs were obtained only for study purposes.

## Results

### Baseline characteristics (Table 1).

**Table 1:** Baseline characteristics.

Patients	N = 201
Women	N = 122 (60.7%)
Men	N = 79 (39.3%)
Age (in years), mean ± SD	53.8 ± 13.6
Disease duration (in years), mean ± SD	6.8 ± 9.8 years
CRP (in mg/l), mean ± SD	10.1 ± 19.6 mg/l
Tender joint count	4.2 ± 5.6
Swollen joint count	1.8 ± 2.8
DAS28, mean ± SD	3.4 ± 1.2
Prednisone^a^	N = 33 (16.4%) Mean dosage: 7.5 ± 8.2 mg
NSAIDs/coxibs^a^	N = 116 (57.7%)
csDMARDs^a^	N = 58 (28.9%)
bDMARDs^a^	N = 27 (13.4%)

*CRP *C-reactive protein, *NSAIDs* non-steroidal anti-inflammatory drugs, *csDMARDs* conventional synthetic disease-modifying antirheumatic drugs, *bDMARDs* biological disease-modifying antirheumatic drugs).

^a^The combination of NSAID/coxibs, csDMARD and bDMARD with prednisone was allowed.

The study enrolled 201 PsA-patients (79 men and 122 women). The mean age was 53.8 ± 13.6 years. The mean disease duration was 6.8 ± 9.8 years and CRP was 10.1 ± 19.6 mg/l. 33 patients were administered prednisone (mean dosage: 7.5 ± 8.2 mg). In addition, 116 subjects were treated with NSAIDs or coxibs, 58 patients received conventional synthetic DMARDs (csDMARDs), and 27 participants biological DMARDs (bDMARDs).

### Influence of age and sex on DXR-BMD, JSD, and Z-score (Fig. 2)

**Figure 2:**
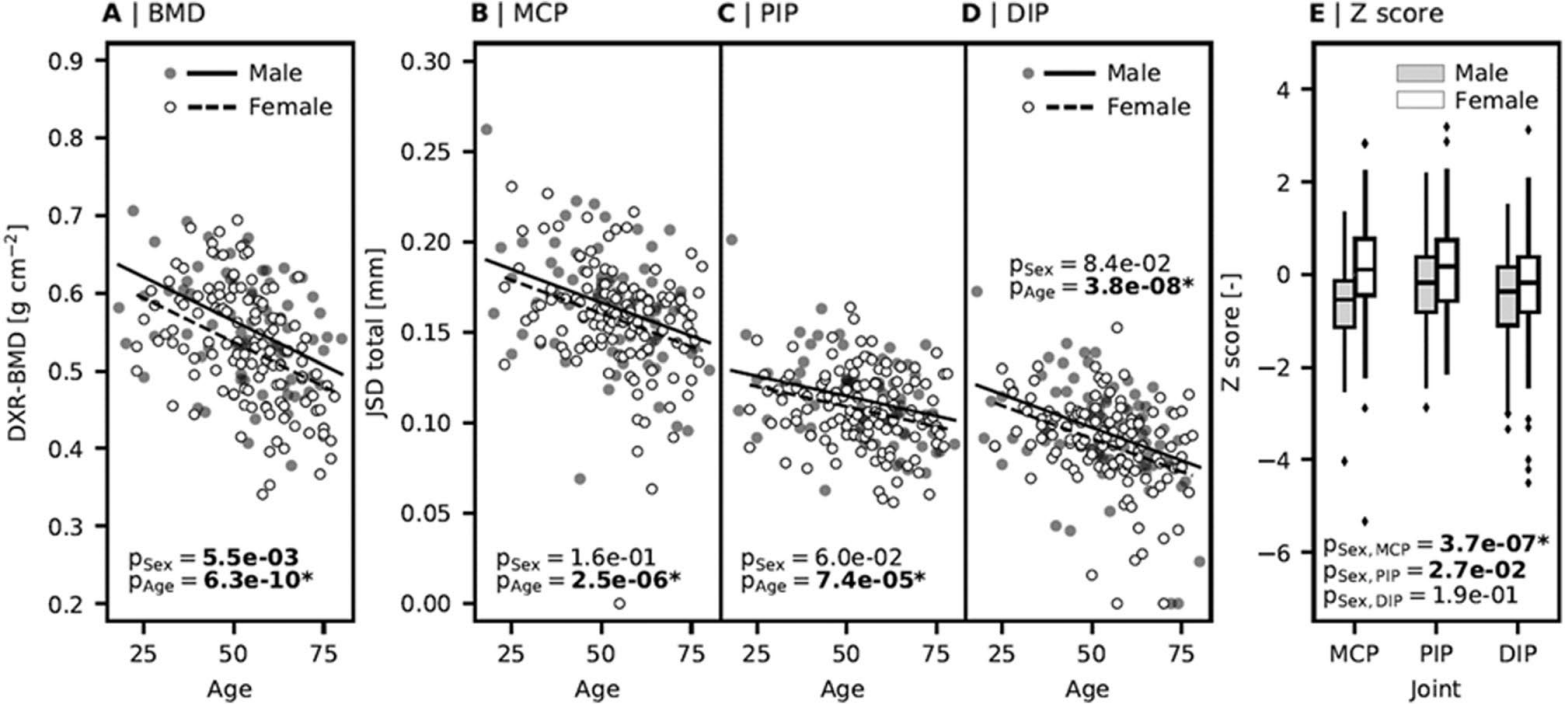
Adjustment of bone mineral density (BMD), measured by digital X-ray radiogrammetry (DXR), finger JSD, and Z-score [metacarpophalangeal joint (MCP), proximal interphalangeal joint (PIP)^1^, distal interphalangeal joint (DIP)] as quantified by computer-aided joint space analysis (CAJSA) regarding age and sex [A: P = 5.5e−03 (P < 0.05), P = 6.3e−10* (P < 0.001); B: P = 1.6e−01 (P = n. s.), P = 2.5e−06* (P < 0.001); C: P = 6.0e−02 (P = n. s.), 7.4e−05* (P < 0.001); D: P = 8.4e−02 (P = n. s.), 3.8e−08* (P < 0.001); E: 3.7e−07* (P < 0.001), P = 2.7e−02 (P < 0.05), P = 1.9e−01 (P = n. s.)].

The linear regression revealed a significant negative influence of age (regression coefficient *β* = − 2.26e−03; *P* < 0.001) and sex (*β* = −2.72e−02;* P* < 0.01) on DXR-BMD. Regarding the interaction of age and JSD, a significant *β of −*7.37e−04 (MCP), −4.43e−04 (PIP), and −7.32e−04 (DIP) was shown.

### Influence of DMARDs- and prednisone treatment, disease duration and disease activity on DXR-BMD, JSD, and Z-score

#### DXR-BMD (Tables 2, 3, 4, 5 and 6, Fig. 3):

**Table 2:** Influence of disease activity score 28 (DAS28) on periarticular mineralization and finger JSD.

	DAS28 < 2.6 inactive PsA, mean ± SD (*N* = 16)	DAS28 > 2.6 active PsA, mean ± SD (*N* = 42)	Difference
**Residuals**			
DXR-BMD in g/cm^2^	−0.0048 ± 0.769	−0.0130 ± 0.728	−0.0082 g/cm^2^
JSD_MCP_ in cm	0.0115 ± 0.0256	0.0010 ± 0.0387	−0.0105 cm
JSD_PIP_ in cm	−0.0069 ± 0.0209	−0.0019 ± 0.0192	0.0050 cm
JSD_DIP_ in cm	0.0096 ± 0.0187	0.0003 ± 0.0251	−0.0093 cm
Z-score_MCP_	0.3395 ± 1.0047	−0.0340 ± 1.3146	−0.3735
Z-score_PIP_	−0.2362 ± 0.9448	−0.0938 ± 0.8846	0.1424
Z-score_DIP_	0.4117 ± 0.8388	−0.0400 ± 1.0665	−0.4517

*DXR-BMD* bone mineral density (g/cm^2^), estimated by digital X-ray radiogrammetry, *JSD* joint space distance (cm) estimated by computer-aided joint space analysis, *MCP* metacarpophalangeal joint, *PIP* proximal-interphalangeal joint, *DIP* distal-interphalangeal joint.

**Table 3:** Effect of swollen joint count (SJC) on periarticular bone loss and JSD.

	SJC = 0, mean ± SD (*N* = 31)	SJC ≥ 1, mean ± SD (*N* = 27)	Difference
**Residuals**			
DXR-BMD in g/cm^2^	−0.0059 ± 0.0680	−0.0242 ± 0.806	−0.0183 g/cm^2^
JSD_MCP_ in cm	0.0016 ± 0.0375	0.0042 ± 0.0350	0.0026 cm
JSD_PIP_ in cm	−0.0048 ± 0.0192	−0.0018 ± 0.0203	0.0030 cm
JSD_DIP_ in cm	0.0042 ± 0.0241	−0.0022 ± 0.0230	−0.0064 cm
Z-score_MCP_	−0.0397 ± 1.3485	0.2301 ± 1.1582	0.2698
Z-score_PIP_	−0.1929 ± 0.8655	0.1629 ± 0.9453	0.3558
Z-score_DIP_	0.1349 ± 1.0297	−0.0620 ± 0.9979	−0.1969

*DXR-BMD* bone mineral density (g/cm^2^), estimated by digital X-ray radiogrammetry, *JSD* joint space distance (cm) estimated by computer-aided joint space analysis, *MCP* metacarpophalangeal joint, *PIP* proximal interphalangeal joint, *DIP* distal interphalangeal joint).

**Table 4:** Effect of tender joint count (TJC) on periarticular bone loss and JSD.

	TJC = 0, mean ± SD (*N* = 17)	TJC ≥ 1, mean ± SD (*N* *= 41)*	Difference
**Residuals**			
DXR-BMD in g/cm^2^	−0.0036 ± 0.0753	−0.0163 ± 0.736	−0.0127 g/cm^2^
JSD_MCP_ in cm	0.0130 ± 0.0258	−0.0003 ± 0.0382	−0.0133 cm
JSD_PIP_ in cm	−0.0061 ± 0.0221	−0.0018 ± 0.0187	0.0043 cm
JSD_DIP_ in cm	0.0081 ± 0.0176	−0.0022 ± 0.0251	−0.0103 cm
Z-score_MCP_	0.3168 ± 0.9517	−0.0397 ± 1.3037	−0.3565
Z-score_PIP_	−0.2095 ± 1.0046	−0.0638 ± 0.8576	0.1457
Z-score_DIP_	0.3509 ± 0.6924	−0.0620 ± 1.0975	−0.4129

*DXR-BMD* bone mineral density (g/cm^2^), estimated by digital X-ray radiogrammetry, *JSD* joint space distance (cm) estimated by computer-aided joint space analysis, *MCP* metacarpophalangeal joint, *PIP* proximal interphalangeal joint, *DIP* distal interphalangeal joint.

**Table 5:** Effect of corticosteroids on periarticular bone loss and JSD.

	No prednisone, mean ± SD (*N* = 168)	Prednisone, mean ± SD (*N* = 33)	Difference
**Residuals**			
DXR-BMD in g/cm^2^	0.0095 ± 0.0676	−0.0288 ± 0.0578	−0.0383 g/cm^2^
JSD_MCP_ in cm	0.0019 ± 0.0282	0.0050 ± 0.044	0.0031 cm
JSD_PIP_ in cm	0.0004 ± 0.0202	−0.0048 ± 0.0247	−0.052 cm
JSD_DIP_ in cm	0.0022 ± 0.0240	0.0042 ± 0.0276	0.0020 cm
Z-score_MCP_	0.0083 ± 0.9712	0.0329 ± 1.2034	0.0246
Z-score_PIP_	0.0825 ± 0.9609	−0.1929 ± 1.0435	−0.2754
Z-score_DIP_	0.1198 ± 1.1145	0.1261 ± 1.2953	0.0063

*DXR-BMD* bone mineral density (g/cm^2^), estimated by digital X-ray radiogrammetry, *JSD* joint space distance (cm) estimated by computer-aided joint space analysis, *MCP* metacarpophalangeal joint, *PIP* proximal interphalangeal joint, *DIP* distal interphalangeal joint.

**Table 6:** Changes of the DXR-BMD and finger JSD as measured by the Z-score dependent on the treatment strategy.

	NSAIDS or coxibs, mean ± SD (*N* = 116)	csDMARDs, mean ± SD (*N* = 58)	bDMARDs, mean ± SD (*N* = 27)	Difference between NSAIDs/coxibs vs. bDMARDs
**Residuals**				
DXR-BMD in g/cm^2^	0.0149 ± 0.0666	−0.0062 ± 0.0658	−0.0231 ± 0.0609	−0.0380 g/cm^2^
JSD_MCP_ in cm	0.0058 ± 0.0236	−0.0011 ± 0.0307	−0.0121 ± 0.0381	−0.0179 cm
JSD_PIP_ in cm	0.0020 ± 0.0213	−0.0033 ± 0.0189	−0.0101 ± 0.0217	−0.0121 cm
JSD_DIP_ in cm	0.0030 ± 0.0242	0.0035 ± 0.0245	−0.0037 ± 0.0253	−0.0067 cm
Z-score_MCP_	0.2217 ± 0.8061	−0.1266 ± 1.0823	−0.2811 ± 1.3292	−0.5028
Z-score_PIP_	0.1504 ± 0.9777	−0.0941 ± 0.8956	−0.4221 ± 1.0326	−0.5725
Z-score_DIP_	0.1392 ± 1.1549	0.0640 ± 1.0865	−0.0215 ± 1.1714	−0.1607

*DXR-BMD* bone mineral density (g/cm^2^), estimated by digital X-ray radiogrammetry, *JSD* joint space distance (cm) estimated by computer-aided joint space analysis, *MCP* metacarpophalangeal joint, *PIP* proximal-interphalangeal joint, *DIP* distal-interphalangeal joint.

**Figure 3:**
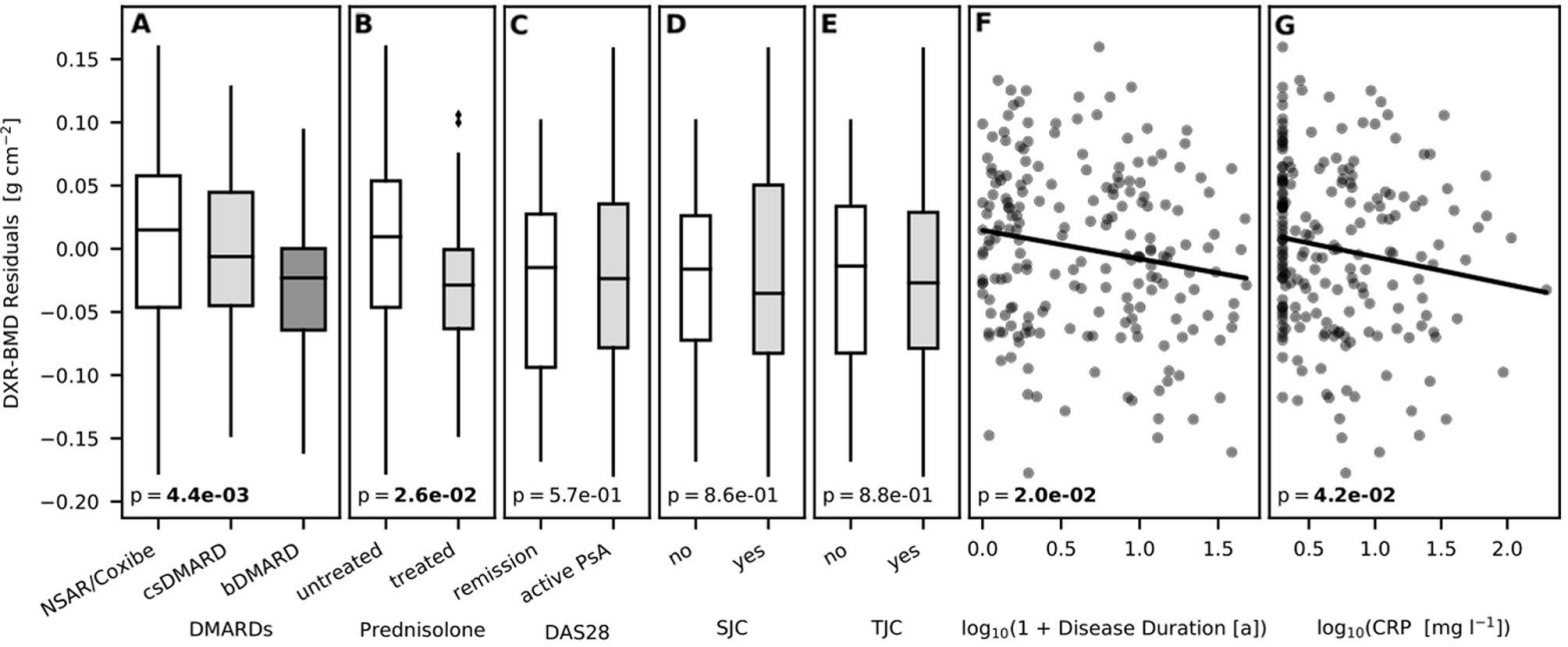
Linear regression model for DMARDs, prednisone, DAS28, SJC, TJC, disease duration, and CRP as independent variables of bone mineral density (DXR-BMD) after the correction of age and sex [A: P = 4.4e−03 (P < 0.05); B: P = 2.6e−02 (P < 0.05); C: P = 5.7e−01 (P = n. s.); D: P = 8.6e−01 (P = n. s.); E: P = 8.8e−01 (P = n. s.); F: P = 2.0e−02 (P < 0.05); G: P = 4.2e−02 (P < 0.05)].

Regarding *disease duration,* there was a significant reduction of the DXR-BMD (difference: −0.0036 g/cm^2^) between a disease duration less than two years and more than 10 years.

*DAS28* revealed a non-significant difference (−0.0082 g/cm^2^) for active PsA. A similar result was quantified for *TJC* (difference: −0.0127 g/cm^2^) and *SJC* (difference: −0.0183 g/cm^2^). Concerning *CRP levels*, a significant change was evaluated for DXR-BMD.

An equivalent result was observed for *treatment* with *prednisone* on DXR-BMD (difference: −0.0383 g/cm^2^). Patients treated with bDMARDs presented a significant lower DXR-BMD (difference: −0.0380 g/cm^2^), compared to patients treated with NSAIDs/coxibs.

#### JSD (Tables 2, 3, 4, 5 and 6, Fig. 4)

**Figure 4:**
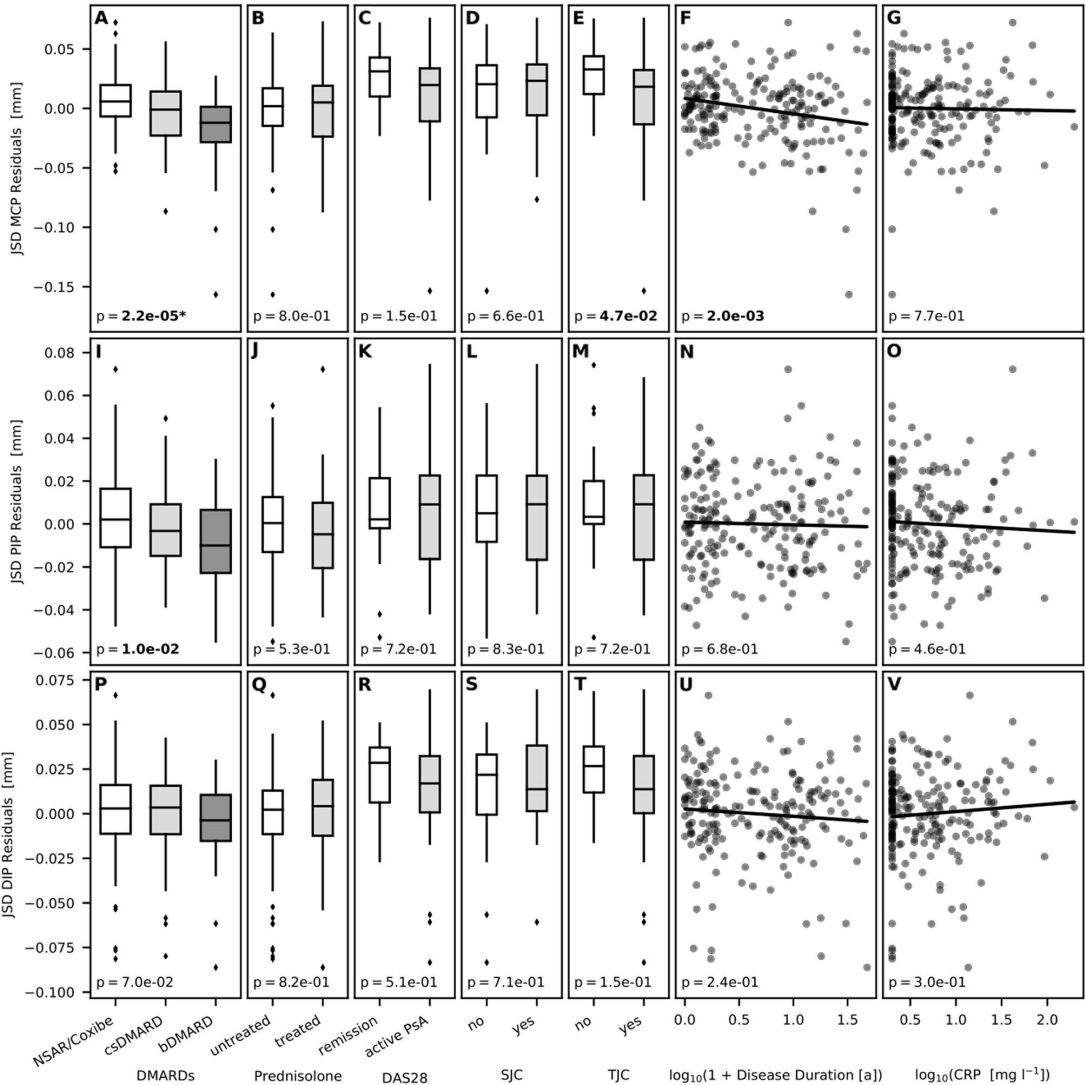
Linear regression model for DMARDs, prednisone, DAS28, SJC, TJC, disease duration, and CRP as independent variables on JSD [metacarpophalangeal joint (MCP), proximal interphalangeal joint (PIP), distal interphalangeal joint (DIP)] after the correction of age and sex [A: P = 2.2e−05 (P < 0.001); B: P = 8.0e−01 (P = n. s.); C: P = 1.5e−01 (P = n. s.); D: P = 6.6e−01 (P = n. s.); E: P = 4.7e−02 (P < 0.05); F: P = 2.0e−03 (P < 0.05); G: P = 7.7e−01 (P = n. s.); I: P = 1.0e−02 (p < 0.05); J: P = 5.3e−01 (P = n.s.); K: P = 7.2e−01 (P = n.s.); L: P = 8.3e−01 (P = n.s.); M: P = 7.2e−01 (P = n.s.); N: P = 6.8e−01 (P = n.s.); O: P = 4.6e−01 (P = n.s.); P: P = 7.0e–02 (P = n.s.); Q: P = 8.2e−01 (P = n.s.); R: P = 5.1e−01 (P = n.s.); S: P = 7.1e−01 (P = n.s.); T: P = 1.5e−01 (P = n.s.); U: P = 2.4e−01 (P = n.s.); V: P = 3.0e−01 (P = n.s.)].

*Disease duration, SJC, TJC, DAS28* and *CRP* showed no significant association to all JSD-parameters in the regression analysis. This also applies to *prednisone,* showing no significant impact on finger JSD. Furthermore, linear regression analysis revealed a significant influence of *DMARDs* on JSD_MCP_ (difference: −0.0179 cm) and JSD_PIP_ (difference: −0.0121 cm). DMARDs revealed no significant linear regression analysis to JSD_DIP_.

#### Z-score (Tables 2, 3, 4, 5 and 6, Fig. 5)

**Figure 5:**
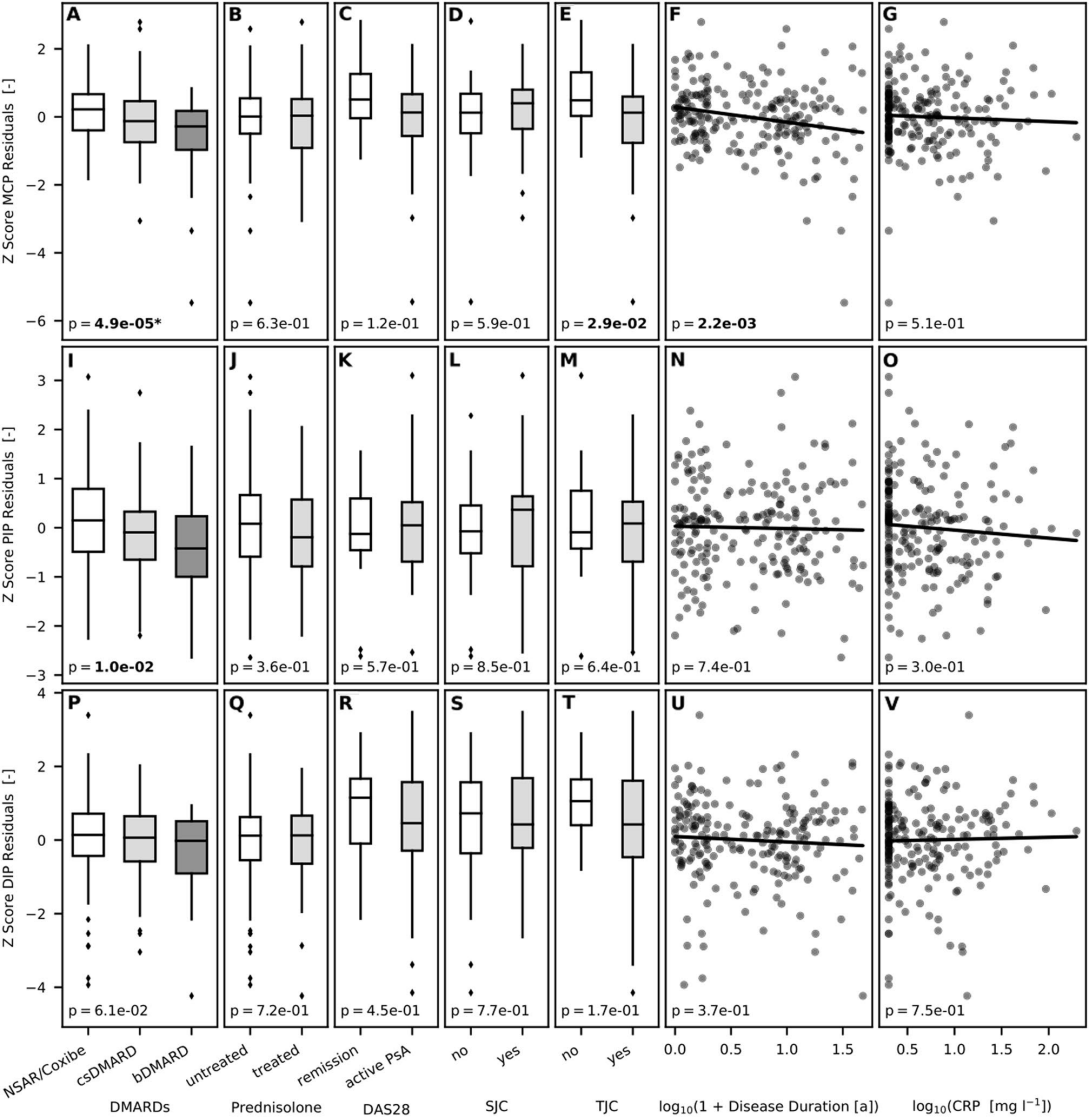
Linear regression model for DMARDs, prednisone, DAS28, SJC, TJC, disease duration, and CRP as independent variables on the Z-score [metacarpophalangeal joint (MCP), proximal interphalangeal joint (PIP), distal interphalangeal joint (DIP)] after the correction of age and sex [A: P = 4.9e–05 (P < 0.001); B: P = 6.3e−01 (P = n. s.); C: P = 1.2e−01 (P = n.s.); D: P = 5.9e−01 (P = n.s.); E: P = 2.9e−01 (P < 0.05); F: P = 2.2e−03 (P < 0.05); G: P = 5.1e−01 (P = n.s.); I: P = 1.0e−02 (P < 0.05); J: P = 3.6e−01 (p = n. s.); K: P = 5.7e−01 (P = n.s.); L: P = 8.5e−01 (P = n.s.); M: P = 6.4e−01 (P = n.s.); N: P = 7.4e−01 (P = n.s.); O: P = 3.0e−01 (P = n.s.); P: P = 6.1e−02 (P = n.s.); Q: P = 7.2e−01 (P = n.s.); R: P = 4.5e−01 (P = n.s.); S: P = 7.7e−01 (P = n.s.); T: P = 1.7e−01 (P = n.s.); U: P = 3.7e−01 (P = n.s.); V: P = 7.5e−01 (P = n.s.)].

No association was found between the Z-score and the treatment with *disease duration*, *SJC, TJC, DAS28, CRP* and *prednisone*.

In accordance with JSD, the Z-score of MCP- and PIP-joints was influenced by *bDMARDs* with a reduction of the Z-score_MCP_ (difference: −0.5028) and Z-score_PIP_ (difference: −0.5725), whereas the Z-score_DIP_ was not significantly reduced.

## Discussion

In contrast to the significant improvement in the understanding of the pathogenesis and treatment of PsA in the last ten years, there is only little known regarding the impact of clinical features on structural and radiological damage. To close this gap, the aim of our retrospective study was to quantify clinical parameters which influence periarticular mineralization of the metacarpal bones and finger JSD in PsA-patients.

In our study, a decrease in DXR-BMD was found for both disease duration and elevated CRP. In addition, prednisone use was associated with a decreased DXR-BMD. However, these parameters did not have an impact on JSD and Z-score. Furthermore, we demonstrated a decrease in DXR-BMD and JSD_MCP_ as well as JSD_PIP_ under therapy with bDMARDs.

Computer-aided techniques including DXR and CAJSA serve as innovative tools in the quantification of radiological damage^5,6,13^. The computer-based structural analysis of hand radiographs offers significant advantages in the quantification of radiographic damage in inflammatory arthritis (e. g. RA and PsA) in comparison with conventional scoring of hand radiographs^6,13–15^. Mainly, the computer-based measurement of DXR-BMD (coefficient of variation: 0.13–1.50%) and CAJSA-JSD (coefficient of variation: 0.38–0.66%) provide a high reproducibility, compared to standard scoring methods for the detection of radiographic damage (e. g. reproducibility of the van der Heijde modification of the Sharp Score: 1.8–3.8%)^16–18^. Therefore, detailed changes of bone structure and JSD are noticeable, especially with regard to the detection of treatment effects, and represent markers for structural damage^13–15^.

The present study evaluated the influence of clinical features such as disease duration, inflammatory activity, prednisone use, and other different treatment strategies on structural parameters of the hands as measured by DXR-BMD and finger JSD in PsA-patients.

In our study, PsA-patients with a disease duration of more than ten years showed a significantly reduced DXR-BMD, JSD_MCP_ and Z-Score_MCP_. This is in accordance with previous data, which revealed an increase of erosive bone changes in PsA, depending on disease duration as measured with high-resolution peripheral quantitative computed-tomography (HR-pQCT)^19^. In this context, the results of our study revealed a significant influence of age on DXR-BMD and finger JSD in PsA patients which has also been shown in healthy subjects^20–22^. Simon et al. reported a lower volumetric articular BMD of the MCP-joints, measured by HR-pQCT in PsA-patients, compared with healthy subjects^23^. In addition, patients with RA showed a significantly reduced DXR-BMD (− 20.7%) and JSD of MCP-joints (− 18.9%) versus a healthy control group^12,24^. Sex had an additional impact on DXR-BMD, whereas JSD was not influenced. As demonstrated before, the DXR-BMD in healthy women is significantly lower (−12.8%) versus healthy men^20^.

Inflammatory activity is strongly associated with radiographic damage in PsA^25^. Regarding inflammatory activity as measured by CRP levels, PsA-patients with increased CRP values showed a reduced DXR-BMD. The linear regression models yielded a significant impact of CRP on DXR-BMD. Furthermore, Wu et al. reported a significant bone loss of the second and third metacarpal bone head, quantified by HR-pQCT in association with elevated CRP values in PsA-patients^26^. These results highlight the strong interaction of inflammatory activity and demineralization of metacarpal bones in inflammatory arthritis which was also published for RA^27,28^. Additionally, CRP served as an independent predictor of radiographic progression in PsA^29^. At the time of initial diagnosis, elevated CRP levels were associated with new erosions as a marker for radiographic progression^30^.

We demonstrated, that treatment with prednisone was associated with a significant reduction of DXR-BMD ( -0.0383 g/cm²). A negative correlation between the change of DXR-BMD over 24 months and the use of corticosteroids (r = −0.27; P < 0.005) was also observed by Hoff et al.^10^. These results indicate that corticosteroids can lead to a demineralization of metacarpal bones. The JSD-parameters in our study revealed no significant changes in association with prednisone treatment. Bond et al. showed an increase of radiological damage, assessed with the Steinbrocker staging system under corticosteroids^31^, where the Steinbrocker method represented a composite score including the quantification of periarticular demineralisations, erosions, joint space narrowing and ankyloses.

Recently published studies showed periarticular metacarpal demineralization^6,9,10,19^ as well as metacarpal bone loss dependent on radiographic damage quantified by Psoriatic Arthritis modified van der Heijde Sharp Score or Psoriatic Arthritis Ratingen Score in patients with PsA^6^. Additionally, finger joint space narrowing represented a common radiographic hallmark for structural damage in PsA. In summary, both parameters reflected PsA-associated radiographic damage. In the present study, treatment with bDMARDs resulted in a significant lower DXR-BMD (−0.0380 g/cm^2^), JSD_MCP_ (−0.0179 cm), JSD_PIP_ (−0.0121 cm), Z-score_MCP_ (−0.5028) and Z-score_PIP_ (−0.5725) compared to csDMARDs and NSAIDs. Concerning DXR-BMD and CAJSA-JSD as radiographic markers of damage and disease severity^5,8^, bDMARDs-treated patients in our study showed more structural damage, which is explainable with the advanced course of the disease.

As a result, patients with an accentuated radiographic damage should be treated earlier with bDMARDs to avoid radiographic structural damage. In this case, the use of csDMARDs should be critically questioned, whereas bDMARDs revealed also higher response rates and reduced radiographic progression in PsA^32^. Radiographic damage and progression correlated with the Health Assessment Questionnaire (HAQ) as outcome marker for disability^33^.

Our study is potentially limited by the cross-sectional design which showed an association of the clinical characteristics of PsA-patients in the mineralisation of the metacarpal bones and the finger joint space width. Further investigations are needed to verify these results in a longitudinal prospective study design. However, the present study offered the first insights of the influence of clinical features on DXR-BMD and JSD as surrogate markers of radiographic damage in PsA patients.

## Conclusion

In conclusion, metacarpal BMD measured by DXR and finger JSD quantified by CAJSA are established markers in the quantification of radiographic damage in PsA-patients. Metacarpal BMD was more influenced by clinical factors like disease duration, inflammatory activity, prednisone, and DMARDs compared to finger JSD which was significantly reduced by DMARDs. In this context, DXR-BMD and JSD represent radiographic damage under different aspects.

## Data Availability

The datasets used and/or analysed during the current study are available from the corresponding author on reasonable request.

## Abbreviations

bDMARDs: Biological disease-modifying anti-rheumatic drugs
CAJSA: Computer-aided joint space analysis
CRP: C-reactive protein
csDMARDs: Conventional synthetic disease-modifying antirheumatic drugs
DIP: Distal interphalangeal joint
DAS28: Disease Activity Score 28
DXR: Digital X-ray radiogrammetry
DXR-BMD: Bone mineral density (g/cm^2^) estimated by DXR
HR-pQCT: High-resolution peripheral quantitative computed-tomography
JSD: Joint space distance
MCP: Metacarpophalangeal joint
NSAIDs: Non-steroidal anti-inflammatory drugs
n.s.: Not significant
PIP: Proximal interphalangeal joint
PsA: Psoriatic arthritis
SD: Standard deviation
SJC: Swollen joint count
TJC: Tender joint count
